# Development of Processes and Catalysts for Biomass to Hydrocarbons at Moderate Conditions: A Comprehensive Review

**DOI:** 10.3390/nano13212845

**Published:** 2023-10-27

**Authors:** Reem Shomal, Ying Zheng

**Affiliations:** Department of Chemical and Biochemical Engineering, Western University, 1150 Richmond Street, London, ON N6A 3K7, Canada; rshomal@uwo.ca

**Keywords:** bio-oil upgrading, hydrodeoxygenation, catalysts, coking, regeneration, eco-friendly energy alternatives

## Abstract

This comprehensive review explores recent catalyst advancements for the hydrodeoxygenation (HDO) of aromatic oxygenates derived from lignin, with a specific focus on the selective production of valuable aromatics under moderate reaction conditions. It addresses critical challenges in bio-crude oil upgrading, encompassing issues related to catalyst deactivation from coking, methods to mitigate deactivation, and techniques for catalyst regeneration. The study investigates various oxygenates found in bio-crude oil, such as phenol, guaiacol, anisole, and catechol, elucidating their conversion pathways during HDO. The review emphasizes the paramount importance of selectively generating arenes by directly cleaving C–O bonds while avoiding unwanted ring hydrogenation pathways. A comparative analysis of different bio-crude oil upgrading processes underscores the need to enhance biofuel quality for practical applications. Additionally, the review focuses on catalyst design for HDO. It compares six major catalyst categories, including metal sulfides, transition metals, metal phosphides, nitrides, carbides, and oxides, to provide insights for efficient bio-crude oil upgrading toward sustainable and eco-friendly energy alternatives.

## 1. Introduction

The increasing awareness of environmental preservation, coupled with rising global energy demand, has shifted focus towards renewable energy sources [1]. This shift is driven by the volatility of fossil fuel prices and the imminent depletion of these finite resources, emphasizing the urgent need for reliable and sustainable energy alternatives [2]. Unlike conventional fuels, ideal fuel substitutes should not only minimize emissions of harmful gases but also represent qualities such as renewability, non-toxicity, and biodegradability [3,4]. Among many potential alternative energies, biofuels, wind, and solar power stand out the most [5]. In this developing energy scenario, biomass is emerging as an environmentally friendly energy alternative [5,6].

Efficiently converting biomass into useful, low-carbon-footprint chemicals and biofuels stands as an effective strategy to address challenges presented by climate change, the depletion of fossil fuels, and other environmental problems [7,8]

Among thermochemical conversion methods, fast pyrolysis stands out as a particularly promising approach to convert lignocellulosic biomass into liquid bio-crude oils [9]. In this method, biomass is heated to temperatures between 400 and 650 °C without the presence of oxygen, causing the material to decompose into liquid bio-crude oil, solid bio-char, and non-condensable gas [10]. While bio-crude oils are produced in an oxygen-free environment, they tend to retain higher oxygen levels due to the composition of the biomass feedstock, setting them apart from fossil fuels and pure hydrocarbons [9,10]. However, the current commercial viability of pyrolysis bio-crude oil is limited due to its less favorable chemical and physical attributes. These challenges encompass the need for preheating bio-crude oil, the potential for injector corrosion, ignition delays, clogging problems, and overall engine instability. To fully harness its potential, it is crucial to significantly reduce the oxygen content in bio-crude oils and carefully control the boiling point fractions, optimizing their ability to blend with or substitute fossil-based transportation fuels [11,12,13,14,15,16].

To date, numerous bio-crude oil upgrading approaches have been explored, as outlined in recent review articles [12,13,14,15]. The upgrading can be achieved using conventional technologies like esterification, hydrogenation, steam reforming, emulsification, and cracking, as well as emerging methods like non-thermal plasma reactions. Among these, hydrodeoxygenation (HDO) is recognized as a practical approach to oxygen removal from bio-crude oil, leading to increased organic phase yield, improved deoxygenation, and reduced catalyst deactivation [16,17,18,19,20,21].

To the best of our knowledge, there is a lack of comprehensive review papers that thoroughly investigate both the chemical and physical methods used in biofuel upgrading, as well as categorize the various catalysts involved in HDO [22,23,24,25]. Therefore, it is imperative to conduct a comprehensive review of the latest methods, evaluating their advantages, limitations, and economic feasibility to identify the most promising approaches for bio-crude oil upgrading. With these considerations in mind, this review seeks to provide an in-depth understanding of the most recent advancements in bio-crude oil upgrading methods. It outlines the pathways for common bio-crude oil model compounds and categorizes the primary catalyst types employed in HDO, while also comparing their respective advantages and drawbacks. Furthermore, the review addresses the prevalent issue of coke formation and explores commonly used methods for catalyst regeneration.

## 2. Properties of Bio-Crude Oil

After pyrolysis, the common oxygenated compounds found in bio-crude oil include acids, aldehydes, alcohols, ketones, esters, phenolics (20–30 wt%) derived from lignin, sugars, furans, and high molecular weight species like oligomers derived from lignin, which are all present in significant amounts but in low concentrations [26], are summarized in Table 1.

Biomass, by definition, encompasses biologically derived and biodegradable materials, excluding peat and petrified substances. Common feedstocks, such as wood, bagasse, rice straw, switchgrass, and wheat straw, are often used in the production of pyrolysis oil [27]. Among the various techniques, fast pyrolysis stands out as one of the most widely adopted technologies. In this process, biomass is subjected to moderate temperatures (around 500 °C) and short residence times (typically seconds) in an oxygen-deprived environment [28]. This results in the rapid depolymerization of biomass, producing pyrolysis vapor, which, upon cooling, condenses into a liquid known as “bio-oil”. In the literature, various terms are used to refer to bio-oil, including “pyrolysis oil”, “bio-crude oil” and “pyrolysis liquid”. However, for the sake of consistency, this review will predominantly use the term “bio-crude oil”.

This bio-crude oil, a dark brown viscous liquid, comprises a mixture of light hydrocarbons and oxygenated compounds, and remarkably, it contains a high liquid content, constituting up to 75 wt%, while generating gas and char as by-products, albeit in lower yields of about 12 wt% and 13 wt%, respectively [29]. These oxygenated compounds encompass acids, aldehydes, alcohols, ketones, and esters; although they are typically present in relatively low concentrations. Moreover, a significant portion, accounting for about 20 to 30 wt%, is composed of phenolic compounds derived from lignin, including substances like phenol, catechol, anisole, and guaiacol, and also includes sugars, furans, and high molecular weight species like oligomers originating from lignin [9,30]. Oligomers are primarily found in bio-crude oil due to the depolymerization process, which entails the breaking of C–O and C–C bonds. This process is followed by subsequent dehydration and repolymerization reactions involving the primary biomass polymers [31]. Most of the typical compounds are summarized in Table 1. 

However, several factors influence the composition and production of bio-crude oil, including the type of feedstock used, moisture content, particle size, the configuration of the pyrolysis reactor, and various pyrolysis conditions like temperature, time, heat transfer rate, condensation efficiency, and char removal efficiency [9]. For instance, lignocellulosic biomass often contains a wide range of alkali and alkaline earth metal compounds such as potassium (K) and sodium (Na), magnesium (Mg), and calcium (Ca). These elements contribute to various characteristics and can promote the production of acetic acid and water. For example, potassium can catalyze the decomposition of liquid compounds by promoting fragmentation over depolymerization reactions, resulting in higher yields of gas, char, and reaction water [32,33].

However, the characteristics of bio-crude oil, including its high viscosity, acidity, and oxygen and water content, result in adverse consequences when used, such as corrosion of metal components, short shelf-life, and diminished heating value [34,35].

Bio-crude oil exhibits substantial differences from crude oil in several critical aspects. Notably, it contains a water content ranging from 15 to 30 wt%, produced from both dehydration reactions occurring during pyrolysis and the moisture present in the initial biomass feedstock. Traditional distillation techniques are often used to eliminate this water content, but they prove ineffective in reducing the elevated water content of bio-crude oil. During distillation, the application of heat triggers rapid polymerization, resulting in the formation of residual solids and unwanted coke, typically in the range of 30–50 wt% [30].

The high oxygen content of around 30–55 wt% notably reduces its heating value of around 16–19 MJ/kg, resulting in an energy density that is less than half that of crude oil of around 40 MJ/kg. Bio-crude oil tends to have a low pH between 2–4, primarily due to the presence of acetic acid and formic acid, which can pose challenges for processing, transportation, and storage equipment. Furthermore, the presence of phenols, olefins, and acids can easily lead to the formation of macromolecules through polymerization, increasing the viscosity and density of biocrude oil and diminishing its fluidity of around 40–100 cP and density 1200 kg/m^3^ [9,33,35,36,37]. Some of the characteristics of heavy petroleum fuel oils and bio-crude oils made through fast pyrolysis are listed in Table 2. Therefore, it is essential to improve the standard of bio-crude oils by minimizing or eliminating these negative characteristics prior to its practical application. 

## 3. Lignin-Derived Aromatic Oxygenates

Compounds containing aromatic oxygenates have served as valuable reference feedstocks in the bio-crude oil upgrading processes in the literature, particularly those primarily derived from lignin. Among these aromatic compounds, m-cresol, anisole, eugenol, and guaiacol have emerged as some of the most popular model feedstocks [38]. 

One of the primary reactions used in this hydrotreatment method to remove oxygen is hydrodeoxygenation (HDO). The procedure also includes additional reactions like transalkylation with acid catalysis, hydrogenolysis of ether bonds (like the breakage of the C_6_H_5_O-CH_3_ bond), as well as aromatic ring hydrogenation. Finally, aliphatic oxygenates, aromatic hydrocarbons, and aliphatic hydrocarbons are produced [39]. In this section, three types of most common oxygenates, phenol, guaiacol, anisole, and catechol, will be discussed in terms of possible mechanism pathways.

### 3.1. Phenol and Alkylated Phenols

**Figure 1:**
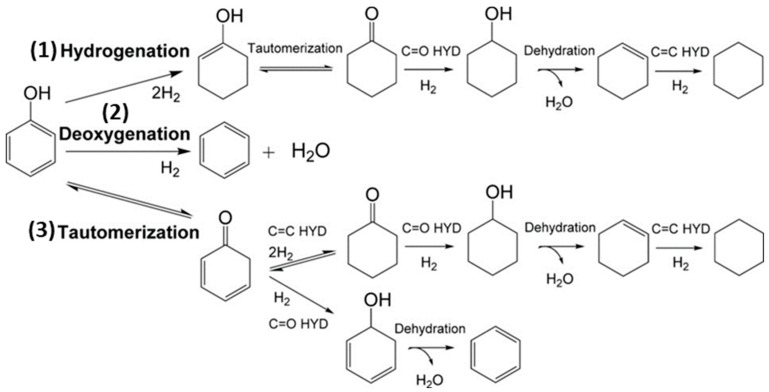
Reaction scheme of three pathways of phenol hydrodeoxygenation, (1) full hydrodeoxygenation, (2) direct deoxygenation, and (3) tautomerization followed by hydrogenation and dehydration [41].

Phenol and its alkylated forms, including cresol and 2-ethylphenol, are the primary phenolic monomers derived from lignin. In the context of hydrodeoxygenation (HDO) aimed at producing arenes and cyclohexane, three main reaction pathways have been proposed, shown in Figure 1 and based on the relevant literature [40].

The first pathway starts with the hydrogenation (HYD) of phenol, leading to the formation of cyclohexanone/cyclohexanol. Subsequent dehydration transforms it into cyclohexene. Later, hydrogenation and dehydrogenation of cyclohexene take place to yield cyclohexane and benzene, respectively. However, there is a lower chance of the isomerization of cyclohexane, which results in methylcyclopentane (MCP), and ring opening can lead to hexane. To avoid H_2_ consumption, it is undesirable to hydrogenate aromatic rings.

The second route involves the direct deoxygenation (DDO) of the C–O bond cleavage, resulting in benzene. It has been proposed that the C–OH cleavage must occur at high temperatures [41]. Due to reported thermodynamic unfavorability regarding the hydrogenation of benzene to cyclohexene, benzene then undergoes a direct conversion to cyclohexane, bypassing cyclohexene in the process.

Additionally, Resasco and co-workers have proposed the final pathway, which begins with the tautomerization of phenol by keto-enol to produce an unstable intermediate called cyclohexadienone [42]. Subsequently, two potential pathways emerge possibly after tautomerization, and the specific pathway depends on the catalyst. Here, we outline two of these pathways’ Equations (1) and (2) [41]:

Phenol+ 4H_2_ ↔ cyclohexane + H_2_O (HYD) (1)

Phenol + H_2_ ↔ benzene + H_2_O (DDO) (2)

In the DDO Equation (2), one involves the hydrogenation of the carbonyl group, leading to the formation of 2,4-cyclohexadienol, which quickly dehydrates into benzene. The other pathway in the HYD Equation (1), entails the hydrogenation of the carbonyl group to yield cyclohexanol, which subsequently undergoes dehydration to produce cyclohexene, followed by a final step of hydrogenation to result in cyclohexane Although each reaction pathway can result in the formation of benzene, high H_2_ pressure can also lead to increased hydrogenation of tautomerization pathway intermediates, which reduces benzene selectivity [41]. Previous research has shown that both the direct ring saturation pathway and the tautomeric pathway display significant selectivity, especially when the hindrance caused by the thermodynamics of cyclohexene dehydrogenation in benzene production is eliminated under low H_2_ pressure [43]. With this understanding, an effective strategy for producing arenes should focus on improving the ability to break C–O bonds or controlling the ring hydrogenation process [44].

### 3.2. Guaiacol and Anisole

**Figure 2:**
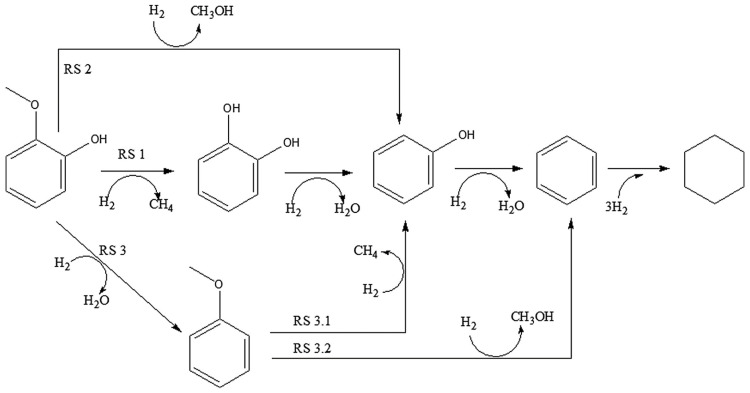
Reaction pathway of guaiacol [46].

Guaiacol is a highly favored model molecule in lignin-derived bio-crude oil studies due to its characteristic hydroxy (C–OH) and methoxy (C–OCH_3_) groups, each contributing to a C–O bond [45]. In the context of the HDO reaction, various pathways for guaiacol conversion have been identified and are summarized in Figure 2 based on the existing literature [46]. In general, three primary mechanisms are observed: (i) demethylation (DME) involving the hydrogenolysis of the C–OCH_3_ bond, resulting in catechol and methane. Catechol typically undergoes deoxygenation under hydrogen pressure [47], which results in phenol and water as by-products; (ii) demethoxylation (DMO) achieved through the direct cleavage of the C–OCH_3_ bond, which produces phenol and methanol; and (iii) hydrogenolysis of the C–OH bond, which generates water and anisole, followed by two subsequent reactions—(1) DME of the C–OCH_3_ bond of the methoxy group present in anisole, producing phenol and methane, and (2) DMO of the C–OCH_3_ bond of anisole, resulting in benzene and methanol. These processes are followed by a three-stage hydrotreatment to yield cyclohexane [48].

**Figure 3:**
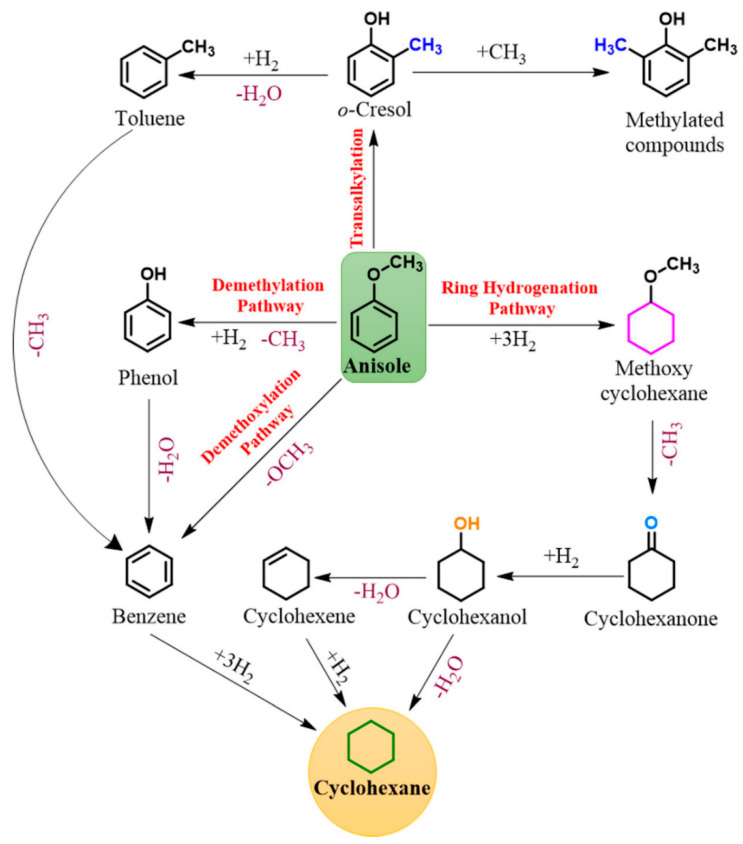
Reaction pathway of Anisole [49].

For anisole, four distinct reaction pathways are evident and shown in Figure 3. Anisole features two distinct C–O (C_aryl_–O–C_alkyl_) bonds: C_aryl_–O with a bond dissociation energy (BDE) of 374.5 kJ mol^–1^ and O–C_alkyl_ with a BDE of 232.6 kJ mol^–1^ [49].

To understand the Anisole pathway, firstly phenol and methane are produced by DME, Benzene and methanol are produced by DMO or the direct deoxygenation route, methoxycyclohexane is produced by hydrogenation of the aromatic ring, and toluene and xylenes are produced as byproducts of methyl group transfer (transalkylation) [50,51]. Due to the DME route’s weaker O–C_alkyl_ bond compared to the DMO route’s stronger C_aryl_–OCH_3_ bond [52], the DME route is kinetically more desirable. Zhu et al. [51] showed that in the presence of an acidic component within the catalyst (acting as an acidic support), the transalkylation pathway becomes the dominant kinetic route. Their findings suggested that catalysts with both acidic and metallic functions facilitate higher rates of both methyl transfer and HDO compared to catalysts with only metallic functions. This results in the production of phenol and BTX products while consuming less hydrogen.

**Figure 4:**
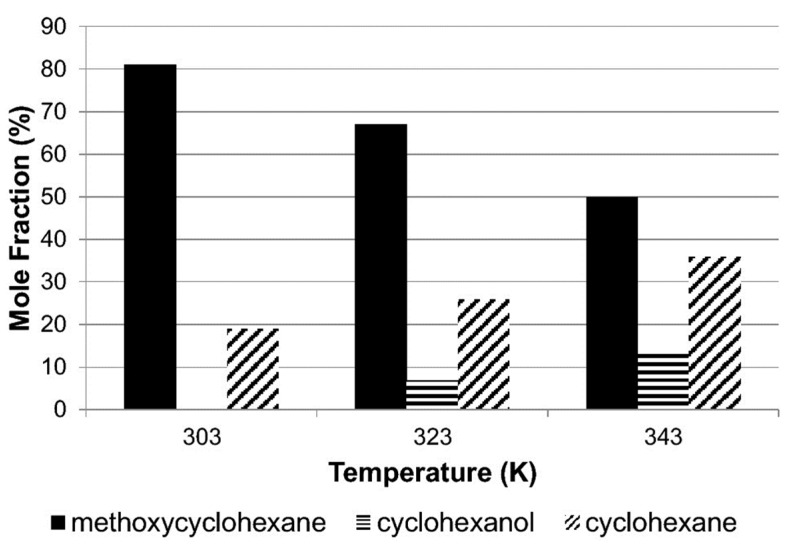
Selectivity achieved from the hydrogenation of anisole as a function of temperature over Rh/Silica [53].

Alshehri et al. [53] conducted a study that emphasized the considerable influence of temperature on anisole’s reaction. This temperature-dependent behavior significantly impacts the reaction pathways and the resulting product selectivity, making it a crucial factor to consider in the process as shown in Figure 4. At lower temperatures, hydro-genation becomes the preferred reaction, leading to higher yields of methoxycyclo-hexane and cyclohexanol. On the other hand, at higher temperatures, hydrogenolysis takes precedence, resulting in increased production of cyclohexane and cyclohexanol. This shift hinges on a crucial step where the C–OCH_3_ bond undergoes cleavage, forming cyclohexane, while simultaneously, the CO–CH_3_ bond undergoes hydrogenolysis, yielding cyclohexanol.

### 3.3. Upgrading Processes of Bio-Crude Oil

**Figure 5:**
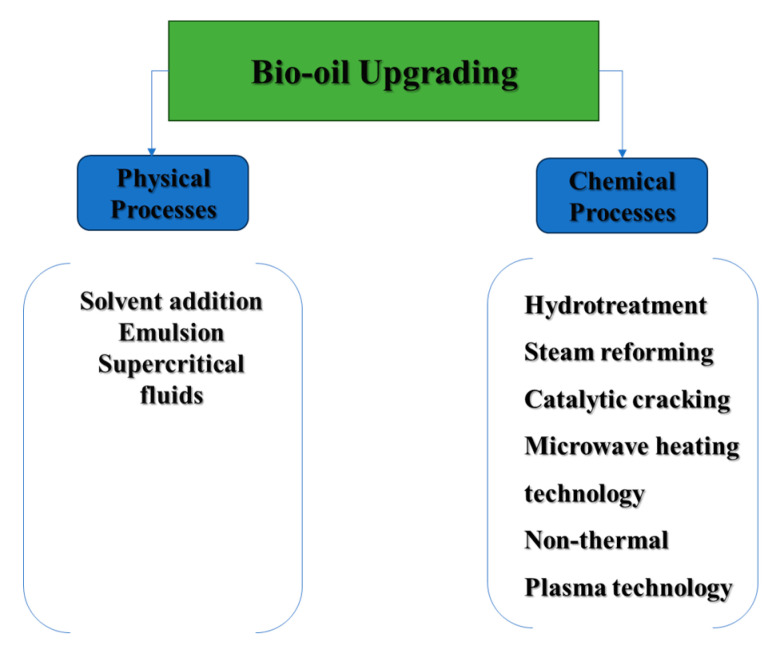
Physical and chemical upgrading processes of bio-crude oil.

Bio-crude oil upgrading has been a subject of extensive research for several years, with the goal of improving its less favorable characteristics to prepare it for further refining [54]. The enhancement process includes both physical and chemical methods, as depicted in Figure 5, which will be further discussed in this review paper. In terms of physical upgrading, efforts are made to address particulate matter by employing filtration techniques. Additionally, blending bio-crude oil with specific additives and solvents is explored to enhance its miscibility and reduce viscosity.

On the other hand, chemical methods involve hydrotreating and hydrocracking of bio-crude oil are designed to eliminate oxygen atoms through hydrogenation, ultimately reducing molecular weights. Additionally, advanced heating technologies such as plasma and microwave heating contribute to the efficiency and precision of the upgrading process.

## 4. Physical Upgrading Processes of Bio-Crude Oil

### 4.1. Solvent Addition

The utilization of bio-crude oil as a conventional transportation fuel faces challenges due to its high viscosity [55]. To mitigate this issue, a simple non-catalytic method, known as solvent addition, has been introduced to improve bio-crude oil. This involves blending polar solvents with the bio-crude oil material to enhance its heating value and reduce viscosity, while also ensuring better homogeneity and preventing phase separation.

Examples of these solvents encompass a range of options such as methanol, ethanol, acetone, ethyl acetate, dichloromethane, water, pentane, hexane, toluene, ether, and ionic liquids [38]. However, the choice of solvents must meet certain criteria, including high selectivity, good solubility, a low boiling point for easy recovery, and ideally, non-toxic properties [31]. Nevertheless, this approach does not effectively tackle the removal of undesirable substances like oxygenates from the bio-crude oil.

Oasmaa et al. [56] conducted research on the impact of adding ethanol, methanol, and iso-propanol to bio-crude oil. Among these alcohols, methanol has proven to be the most effective in enhancing the stability and reducing the viscosity of bio-crude oil, while ethanol has been found to improve its heating efficiency. The addition of methanol to bio-crude oil increases its acidity and reduces viscosity by slowing down polymerization and aging reactions. The rate of viscosity reduction is a critical criterion for assessing effectiveness, and most solvent-based techniques achieve a viscosity reduction of over 70%.

Furthermore, the reduction in viscosity is dependent on various factors related to the addition of solvents to bio-crude oil, such as physical dilution, molecular dilution, esterification, and acetalization reactions between the solvent and the bio-crude oil. These reactions prevent the further growth of long or short-chain organic molecules present in bio-crude oil [54].

However, the use of solvents in the process also leads to alterations in the characteristics of bio-crude oil. For instance, Luo et al. [57] observed a significant 90% reduction in the viscosity of the solvent-enriched liquid phase, even after the removal of the solvent mixture at atmospheric pressure. This suggests that changes in the properties of bio-crude oil extend beyond simple solvent dissolution.

Furthermore, the addition of solvents can affect the pH of bio-crude oil. Mei et al. [58] noted an increase in the pH of bio-crude oil from 2.32 to 3.26 after 35 days of storage with the inclusion of 15 wt% methanol. Another study by Liu et al. [59] investigated the effects of adding acetone on the physicochemical characteristics of the resulting bio-crude oil. The presence of acetone not only increased the pH value of the bio-crude oil but also reduced its water content and viscosity. Moreover, it inhibited aging responses and the formation of new chemicals in the bio-crude oil

Khosravanipour et al. [60]. demonstrated a significant 20-fold reduction in bio-crude oil viscosity by employing methanol in a fluidized bed reactor, highlighting the substantial impact of solvents on the rheological characteristics of bio-crude oil. More recently, Zhu et al. [61] investigated the effects of mixed solvents, compared to using a single solvent, the bio-crude oil had improved viscosity, water content, and pH. Examples of these solvents include acetone, N-dimethylformamide, and methanol. 

Given the cost-effectiveness of certain solvents, this method of bio-crude oil upgrading is regarded as one of the most economically viable approaches to improving fuel properties, enhancing physicochemical stability, and facilitating the long-term storage and transportation of bio-crude oil. Table 3 summarizes the main limitations and advantages of this method. 

### 4.2. Emulsion

Emulsion has emerged as a straightforward physical enhancement technique for bio-crude oil. Unlike other modification procedures, emulsification is a quick and easy method that can reduce the viscosity of bio-crude oil and increase its calorific value, cetane value, and stability. Additionally, it is a cost-effective, hassle-free, and direct method of bio-crude oil upgrading [68]. Table 3 summarizes the main limitations and advantages of this method compared to other methods. This method involves mixing two immiscible liquids by mechanical agitation (stirring) or using a surfactant and co-surfactant [69]. In an emulsion, two phases exist: the dispersed phase (bio-crude oil) and the continuous phase (fossil fuel or conventional hydrocarbon fuel) [70,71].

Various emulsifiers are used in the literature, with the most common being a combination of Span 80 and 60 for emulsifying ether extract bio-crude oil (EEO) and diesel, resulting in a stable emulsion with a calorific value of 44 MJ/enhanced and extended stability for 40 days, ultimately improving the emulsified bio-crude oil’s stability [69]. Additionally, a range of emulsifiers, including Lecithin, Atlox 4914, Lignin, PGO, Lanolin, Hypermer B246, Brij 58, Brij 72, Span 60, Span 80, Span 85, Span 100, and Tween 80, have been utilized for bio-crude oil emulsion [72,73,74]. Selecting an appropriate surfactant is crucial for establishing a stable emulsion system. The hydrophilic-lipophilic balance (HLB) value and chemical characteristics of a surfactant determine its suitability. HLB values range from 0 to 20 and are categorized as hydrophilic or lipophilic. The HLB value reflects the chemical properties and characteristics of the surfactant [75]. To achieve the optimal HLB value, two or more emulsifiers can be combined, although this is not always recommended due to potential changes in temperature stability [76]. 

Optimizing process parameters is essential for successful high-quality bio-crude oil upgrading. This can be accomplished by adjusting factors such as the ratios of diesel-biodiesel to bio-crude oil, emulsifier to surfactant ratios, mixing time, stirring rate, and temperature [31]. In a parametric study by Jiang and Ellis [75], softwood bio-crude oil was enhanced through a biodiesel-assisted emulsion using octanol as a surfactant. Ideal conditions were determined to be at 1200 rpm for a processing time of 15 min and a bio-crude oil/biodiesel ratio. Under these optimal conditions, the viscosity, acidity, and water content of the softwood-derived bio-crude oil were significantly reduced. Successful emulsions were found to work well with bio-crude oil, diesel, and surfactant percentages ranging from 10–50%, 50–90%, and 1–10%, respectively [77,78]. The optimal content of bio-crude oil and surfactant for a stable emulsion bio-crude oil is 10–20 wt% and 4–6 wt%, respectively [77,78]. It is important to highlight that the proportion of bio-crude oil in the emulsion directly impacts its viscosity, with a greater quantity of bio-crude oil leading to increased emulsion viscosity. 

**Figure 6:**
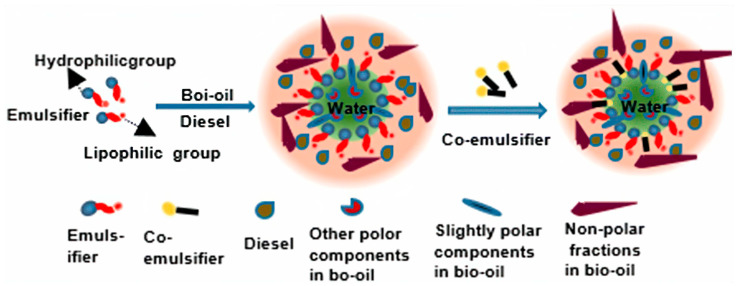
Proposed mechanism model for the emulsion of bio-crude oil and diesel [81].

Another approach is the utilization of emulsifiers and co-emulsifiers such as short-chain or long-chain alcohols, which can facilitate the creation of a stable and uniform system between bio-crude oil and diesel oil, such as methanol, ethanol, and n-butanol binders [79,80]. For example, Jiang et al. [75] conducted research on the thermal stability of bio-crude oil in an emulsion with biodiesel, showing that the mixture of bio-crude oil and biodiesel mixture remained stable for 180 h. And Liu et al. [81], used an emulsifier (a mixture of Span 80 and Twin 80) and a co-emulsifier (2-octanol) for 384 h, with results showing that viscosity, density, heating value, and corrosivity are comparable to diesel oil. Figure 6 depicts the interaction of bio-crude oil and diesel emulsions, as described in the literature [81].

### 4.3. Supercritical Fluids

The use of supercritical fluids (SCFs) represents an innovative approach to bio-crude oil upgrading. In this method, chemicals such as CO_2_, ethanol, methanol, and water have been extensively explored as potential solvents. Supercritical fluids possess unique properties, as they can exhibit both gas-like and liquid-like characteristics under specific temperature and pressure conditions [31,57]. Notably, aside from the fast pyrolysis of biomass, supercritical fluids are applicable for the conversion of biomass into bio-crude oil and the subsequent bio-crude oil upgrading [82,83]. The SCF modification process increases the bio- crude oil’s calorific value while decreasing viscosity. 

The esterification of bio-crude oils occurs under supercritical conditions when acid catalysts and alcohols are used [84]. For example, Lee et al. [85] used a supercritical catalyst fluid with a Ni-based catalyst at temperatures ranging from 250–350 °C for the catalytic upgrading of bio-crude oil obtained from rapid pyrolysis of woody biomass. The MgNiMo/AC catalyst yielded the highest upgraded bio-crude oil yield at 70.5 wt% at 350 °C, with a very low O/C molar ratio of 0.19, a total acid value (TAN) of 6.2 mg KOH/g, and a higher heating value (HHV) of 33.4 MJ/kg. This upgrading process effectively removed undesirable oxygen-containing substances such as carboxylic acids and aldehydes from the bio-crude oil.

However, alcohols are the most popular supercritical fluids that are used to upgrade bio-crude oil. In a study by Prajitno et al. [86] on bio-crude oil upgrading using supercritical ethanol, the need for a catalyst or an external hydrogen source was eliminated. The resulting bio-crude oil exhibited improved properties, including a heating value of 34.1 MJ/kg, a total acid number (TAN) of 4.8 mg KOH/g, and a water content of 1.6 wt%. These values were compared to the raw bio-crude oil, which had a lower heating value (HHV) of 24.3 MJ/kg, a higher TAN of 69.4 mg KOH/g, and a higher water content of 14.0 wt%. The study concluded that the supercritical ethanol method effectively enhances the quality of bio-crude oil. Additionally, the use of water under supercritical conditions has been a viable method for bio-crude oil enhancement. Duan and Savage [82] reported a notable reduction in the total acid number, decreasing from 256 to 25, and an increase in the calorific value of algal bio-crude oil to 43 MJ/kg.

Furthermore, a recent investigation by Shafaghat et al. [64], demonstrated that combining hydrodeoxygenation (HDO) with a supercritical fluid system could significantly enhance deoxygenation and elevate the heating value of bio-crude oil. When methanol was employed as the supercritical fluid, the bio-crude oil’s heating value increased from 12.61 MJ/kg to 24.17 MJ/kg, and approximately 42.71% of the oxygen was successfully removed from the bio-crude oil.

It is crucial to emphasize that while the supercritical fluid method effectively reduces acidity and viscosity while enhancing the energy content of bio-crude oil, it necessitates a reactor capable of withstanding high pressure (usually >150 bar) and materials resistant to corrosion under supercritical conditions, leading to higher operating costs. These conditions are a critical consideration when implementing the supercritical fluid upgrading process for bio-crude oil. The advantages and disadvantages of this process are summarized in Table 3.

## 5. Chemical Processes of Bio-Crude Oil Upgrading

### 5.1. Hydrotreatment

**Figure 7:**
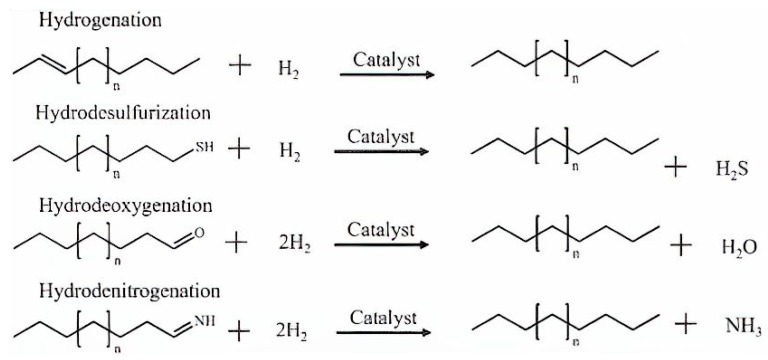
Typical hydrotreating reactions for removing impurities in the presence of an excess of hydrogen [87].

The removal of sulphur, nitrogen, and oxygen heteroatoms is accomplished through the hydrotreating method, which also includes hydrodesulfurization (HDS), hydrodenitrogenation (HDN), and hydrodeoxygenation (HDO). Figure 7 illustrates the stoichiometric chemical reactions equations representing the typical pathways in hydrotreating processes for the elimination of unwanted impurities [87]. In contrast to petroleum oil, bio-crude oils contain minimal amounts of sulphur and nitrogen but have a considerably higher oxygen content of around 33–55%, as shown in Table 2. In an oil refinery, hydrotreatment is a well-established process that is frequently operated at temperatures between 300–450 °C and H_2_ gas pressures to a maximum of 20 MPa. The most commonly used catalysts in hydrotreating operations are NiMo, NiW, and CoMo [88]. 

Hydrotreating methods, including hydrodeoxygenation (HDO), are highly effective at removing oxygen from bio-crude oil [1]. Initially termed “mild hydrotreating”, this process operates at lower temperatures (around 250–300 °C) with the presence of hydrogen and a hydrotreating catalyst to prevent bio-crude oil polymerization. The key enhancement in the HDO process is increased pressure, enhancing hydrogen solubility within bio-crude oil and its diffusion over the catalyst surface. This accelerates reaction rates and reduces coke formation. 

Therefore, cautious selection of heterogeneous catalysts and process conditions is vital. Other variables to be considered, including the type of catalyst, reactor design, temperature, residence time, pressure, solvent, and reactor configuration, have an impact on the yield and properties of improved bio-crude oil produced from HDO [1]. 

**Figure 8:**
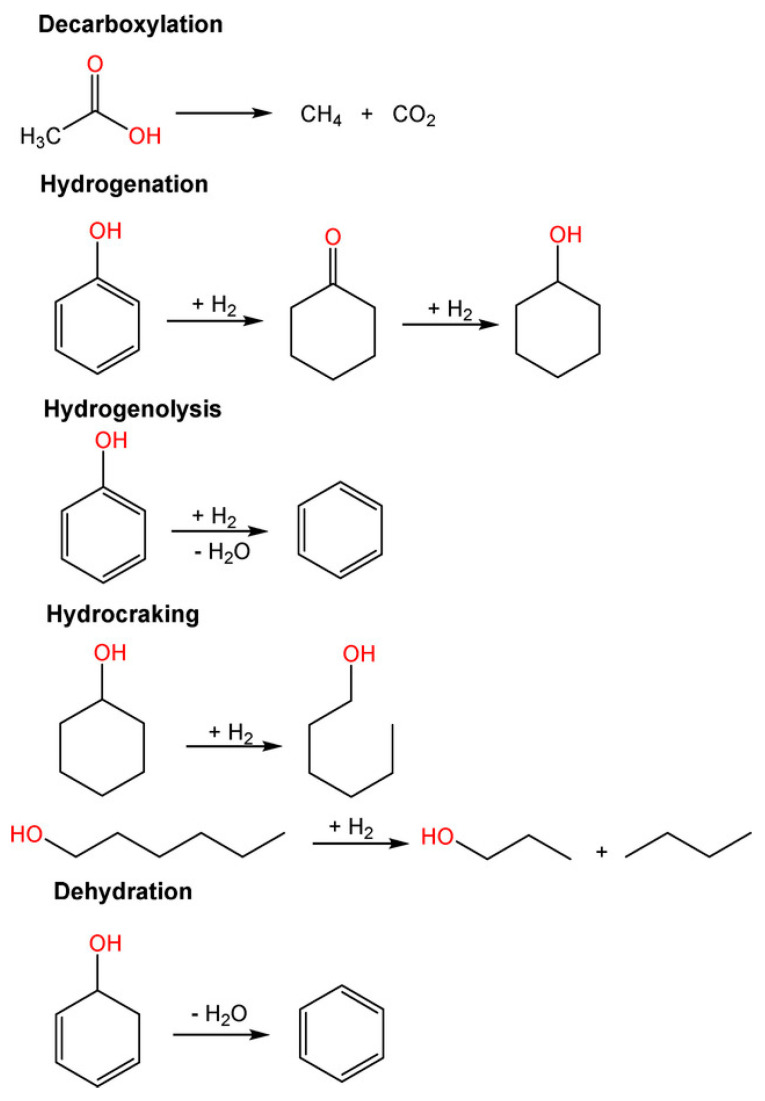
Possible reactions in the HDO process [89].

In addition, HDO reactions include dehydrogenation, condensation-polymerization reactions accompanied with the aid of decarboxylation, then dehydration to convert saturated bonds into unsaturated bonds, isomerization, and hydrogenolysis of C–O and C–O–C bonds to release water molecules [89]. Hydrogenation of carbonyls and C=C bonds or cleavage of C–C bonds occurs at metal active sites during the decarbonylation and decarboxylation pathways. Hydrogenation of aromatics present in bio-crude oil for the duration of HDO should be avoided, as it will consume extra hydrogen and lower the octane number of the product. Consequently, the type of metallic active sites (e.g., monometallic and bimetallic sites) has a huge impact on HDO product selectivity; even the selection of acid support plays an important role in the reaction. The most common pathway of the reaction is presented in Figure 8.

In a study conducted by Elksabi et al. [90], various bio-crude oil samples obtained from switchgrass and horse manure were rapidly pyrolyzed and then subjected to HDO using Pt-, Ru-, and Pd-based catalysts. This HDO process lasted for 4 h at 320 °C and 145 bar. Among these catalysts, Pt/C demonstrated remarkable performance in the HDO of switchgrass-derived bio-crude oil, exhibiting the highest rates of oxygen reduction and carbon retention.

Another study by Elliott and colleagues [91] introduced a two-stage hydrodeoxygenation process for the pyrolysis bio-crude oil. This approach aimed to address the thermal instability of bio-crude oils. In the first stage, mild catalytic hydrogenation at 270 °C and 136 atm was carried out using previously sulfided Co-Mo/Al_2_O_3_ or Ni-Mo/Al_2_O_3_ catalysts. This step primarily targeted the enhancement of the effective H/C ratio in the bio-crude oil, aiming to mitigate the polymerization and coke formation issues associated with high-temperature reactions. The second stage, conducted at elevated temperatures (up to 400 °C) and pressure, focused on removing oxygen from the bio-crude oil in the form of water, resulting in the production of hydrocarbons. This process typically yields 0.4 L of treated oil for every 1 L of bio-crude oil and converts 20–30% of the bio-crude oil’s carbon content into gas-phase carbon, reducing the overall oil production.

While HDO is acknowledged for its effectiveness in bio-crude oil upgrading, outperforming many other techniques for upgrading and separation. The HDO process has two significant drawbacks: a high-pressure requirement for H_2_ and a limited catalyst lifetime due to coke formation [23]. A summary of the advantages and disadvantages of this procedure is provided in Table 3.

### 5.2. Steam Reforming

The steam reforming process holds great promise for generating renewable energy in the form of hydrogen gas and enhancing the value of bio-crude oil. This process is typically carried out in fixed or fluidized bed reactor systems using catalysts made of nickel, cobalt, or noble metals. The reactions occur at atmospheric pressure and high temperatures ranging from 600 to 800 °C [63]. Hydrogen, being a renewable resource, is gaining global attention for its applications in the chemical industry, as a fuel, and as a raw material. Hydrogen-fueled combustion results in the production of water and emits no harmful gases, thereby reducing greenhouse gas emissions [92,93].To gain insights into the fundamentals of steam reforming, various model bio-crude oil molecules, including acetic acid, acetone, phenol, and glycerol, have been studied. Using steam reforming, Lan et al. [94] successfully converted bio-crude oil derived from the fast pyrolysis of rice husks into hydrogen. They achieved this at a liquid hourly space velocity of 0.5 to 1 h, a steam-to-carbon ratio of 15 to 20, and a temperature between 700 and 800 °C, yielding a maximum hydrogen yield of 75.88%.

The process is influenced by variables such as retention time, steam-to-carbon ratio, and reaction temperature. For instance, Mei et al. [95] reported that using a carbon-to-steam ratio of 5:1 and steam reforming of m-cresol, a bio-crude oil model compound, at 850 °C, produced a maximum hydrogen yield of 79.8%. On the other hand, Trane-Restrup et al. [96] demonstrated that increasing the steam-to-carbon ratio between 1.6 and 8.3 enhanced hydrogen output and conversion. Valle et al. [97] found that a carbon ratio of 6 resulted in the highest hydrogen output, ranging from 87% to 93%.

While the production of hydrogen from bio-crude oil through steam reforming is considered feasible, there are still areas that require further investigation, such as catalyst reactivity and stability, susceptibility to coking, and cost considerations. It is worth noting that steam reforming of the aqueous component of raw bio-crude oil produces higher levels of CO and CO_2_, necessitates higher reaction temperatures, and leads to more significant carbonaceous deposits. As a result, this process results in increased catalyst deactivation within the context of a more complex reaction network. These challenges are more pronounced when compared to the reforming of simplified model bio-crude oil compounds [98]. An alternative method for converting bio-crude oil into syngas is bio-crude oil steam gasification. This process operates at temperatures between 800 °C and 1400 °C, in the presence or absence of catalysts. However, it produces syngas with a composition similar to that obtained from biomass gasification, albeit with lower hydrogen yield and energy efficiency compared to steam reforming [99]. The advantages and disadvantages of the process are summarized in Table 3.

### 5.3. Catalytic Hydrocracking and Cracking

Hydrocracking and catalytic cracking are processes designed to break down larger hydrocarbon molecules into smaller hydrocarbon molecules, and often involve subsequent hydrogenation. In hydrocracking, the bio-crude oil is subjected to high temperatures (usually above 350 °C) and high hydrogen pressures (ranging from 0.7 to 21 MPa) in the presence of a heterogeneous catalyst. Similarly, catalytic cracking involves the use of a catalyst to break down the complex organic molecules and also requires high temperatures and typically operates at temperatures above 350 °C without hydrogen source [100]. 

**Figure 9:**
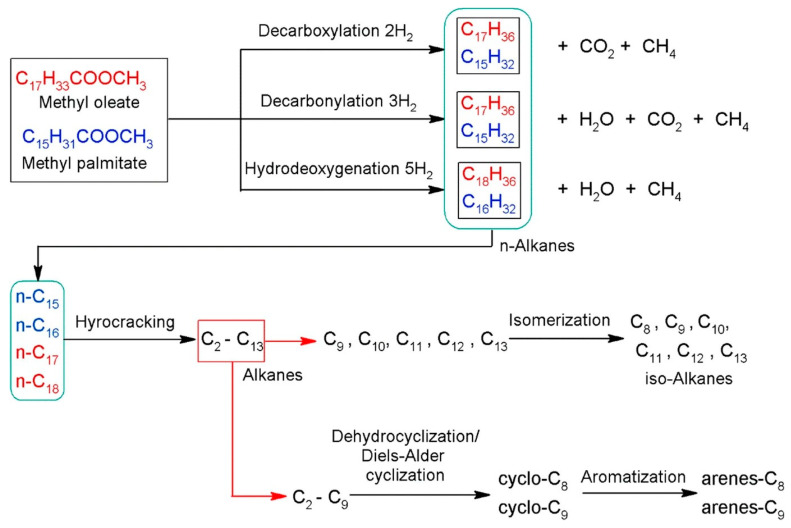
Reaction pathways for the hydrodeoxygenation and hydrocracking of methyl oleate and methyl palmitate [102].

The hydrocracking process comprises two main types of reactions: pre-treating and cracking. During the pre-treating reaction, sulfur, nitrogen, organic metal compounds, halides, and oxygen are removed, leading to an increase in the H/C ratio and preserving hydrocracking catalyst activity and maintaining the desired yield structure. The cracking reaction involves coke formation, isomerization, aromatic saturation, polynuclear aromatic formation, and cracking. To produce high-quality fuel, hydrogen is added to a carbon–carbon double bond after the carbon–carbon single bond is broken. Given that all hydrocracking processes involve contact with hydrogen at pressures above 7.0 MPa and temperatures up to 470 °C, the roles of hydrogen and temperature are critical [101]. Figure 9 depicts the overall pretreating and hydrocracking reaction processes. 

For a conventional hydrocracking catalyst to be effective, it must possess both acidic sites and hydrogenation-dehydrogenation sites as active sites. Cracking processes occur at the acidic sites, while hydrogen transfer is initiated at the hydrogenation-dehydrogenation sites. Acidic sites with high activity tend to accumulate coke, leading to catalyst deactivation. Therefore, achieving a proper balance between these two types of activity sites is essential to manufacture an effective hydrocracking catalyst.

Tanneru and Steele [65], investigated the influence of reaction temperature, hydrogen pressure, residence time, and catalyst type on the hydrocracking of bio-crude oil derived from fast pyrolysis of pinewood chips, aiming to convert it into hydrocarbons. The resulting hydrocarbon mixture had the following properties: 0.9 g/mL density, 1.8 cSt viscosity, 43.6 MJ/kg higher heating value (HHV), 0.5 wt% oxygen content, and 0.3 mg KOH/g total acid number (TAN). Hydrocracking was effective in producing lighter components. However, it is worth noting that achieving this transformation required high operating temperatures and elevated H_2_ pressure, potentially leading to increased overall process costs. The specific limitations of this process are summarized in Table 3.

### 5.4. Novel Approaches for Bio-Crude Oil Upgrading

There are two advanced hydrodeoxygenation (HDO) technologies that aim to improve the process by reducing the need for high H_2_ partial pressures and using milder reaction conditions.

One of these innovative methods is plasma-assisted HDO. In this method, an electric field ionizes a gas to produce plasma, a partially ionized gas containing high-energy species such as ions, electrons, and radicals [103]. Ideally, the electrical energy required for this process can be produced using renewable sources such as solar or wind energy. Unlike traditional methods that use thermal energy to activate the HDO reaction, plasma-assisted HDO uses electrical energy to ionize hydrogen gas and initiate HDO at room temperature and atmospheric pressure. This eliminates the need for expensive high-pressure and high-temperature equipment and significantly reduces carbon dioxide emissions compared to traditional thermal HDO methods [104]. Electrons with an energy range of 1 to 10 eV play a key role in this process. When two reacting molecules collide, electrons transfer their energy to the collision, breaking chemical bonds and creating free radicals. These radicals can recombine to produce lighter molecules [105]. The collision of free electrons and bio-crude oil molecules in the upgrading process breaks chemical bonds (such as C–H, C–O, and O–H) and forms active radicals that can recombine to produce new products [106].

Another method to supply the required hydrogen without relying on external sources is the modification of the aqueous phase of pyrolysis oils, which aims to reduce overall costs and dependence on external hydrogen sources [106]. However, the amount of hydrogen produced using this method is often insufficient for complete upgrading. However, there are new approaches to in situ hydrogen production include plasma cracking of methyl groups produced during the demethylation reaction, which can be used for hydrodeoxygenation or hydrocracking reactions [103].

Taghvaei et al. [107] introduced an HDO reaction with in situ hydrogen production in pulsed corona plasma and showed promising results in carrying out the HDO upgrading process of bio-crude oil at low temperature and atmospheric pressure, while raising concerns about the high consumption of hydrogen gas. While another study by Khalifa et al. [108,109], investigated an efficient in situ conversion of methane in HDO bio-crude oil to hydrogen using a coaxial cylinder dielectric barrier discharge (DBD) plasma. This study demonstrated that extra-pure H_2_ production and a maximum CH_4_ conversion of 100% could be achieved with the packed plasma system at CH_4_ flow rates of up to 20 mL/min. 

Another promising technique involves the use of microwave heating to accelerate the HDO reaction. Although oil interacts relatively little with microwaves, the oxygenated molecules in bio-crude oil show a higher interaction at polar sites. Because polar compounds in pyrolysis oils are potent microwave acceptors, this local interaction generates thermal energy at the location where C–O bond cleavage occurs [66,110]. In particular, many of the catalysts and oxygenated compounds in the oils often used in HDO processes are polar materials and act effectively as microwave-to-heat converters. Due to these aspects, microwave heating is an alternative to the traditional HDO process. However, it is important to note that there are no studies on bio-crude oil upgrading using microwave heating systems and this process has not been extensively investigated in the literature but shows a promising pathway for HDO in the future.

## 6. Catalysts Designed for Hydrodeoxygenation of Lignin-Derived Oxygenates

In this section, we discuss recent developments in selective hydrodeoxygenation catalysts, concentrating mainly on phenol and guaiacol, which are the most basic lignin degradation products. A crucial step in the HDO process is the cleavage of C–OH bonds, and there are two general reaction pathways for generating cycloalkanes and arenes from lignin-derived oxygens [25]. These routes are: (a) hydrogenation of the aromatic ring followed by deoxygenation of the alcohol to form the cycloalkane and (b) direct deoxygenation to arenes by cleavage of C–OH bonds.

Most of the research in this field has been aimed at promoting DDO pathways to prevent aromatic ring hydrogenation. By prioritizing the DDO route, hydrogen consumption can be reduced, resulting in lower operating pressures and costs. In addition, this approach leads to the production of more valuable aromatic compounds, especially benzene, toluene, and xylene (BTX) [111].

### 6.1. Designed Catalysts for Lignin-Derived Oxygenates

This research paper emphasizes the catalytic properties of the hydrodeoxygenation process. Even though HDO reactions are very exothermic and have favorable thermodynamics, they are rarely initiated without the aid of a catalyst and high temperatures are required to continue at a significant rate [9].

Several important factors must be considered in catalyst design for HDO. An ideal HDO catalyst should have selectivity for hydrogen molecules and oxygen, and exhibit activity in hydrogenation reactions Notably, industrial lignin undergoes a wide range of chemical reactions at temperatures of 380–430 °C, a including cleavage of interunit bonds, release of oxygen, ring hydrogenation, including the removal of alkyl and methoxyl moieties [23]. Catalysts should exhibit high activity for C–O–C and C–C bond hydrolysis and/or cleavage, and remain low in activity for ring hydrogenation. Furthermore, a reasonable choice selectivity for specific aromatic compounds or chemical compositions is important to facilitate efficient refining. 

Catalysts must also resist deactivation, especially because of coking, which is a major cause of catalytic deactivation in HDO in biofuels. Operation at low to moderate temperatures between 200 and 300 °C is recommended to reduce coking [112]. Furthermore, the acidity of the catalyst must be sufficient to promote isomerization and reduce fragmentation, to maximize product yield. From an economic and environmental point of view, it is important to minimize the unnecessary consumption of H_2_ during HDO, and this is sensitive to the catalyst composition [111]. Catalytic hydrogen consumption in the HDO process deserves further attention to accelerate the commercialization of bio- crude oil upgrading by the catalytic method. 

The technological deficiencies in the HDO process for bio-crude oil pose additional difficulties owing to the poor quality of bio-crude oil (characterized by high oxygen content, impurities, and coking tendencies), as well as the need for high-pressure and high-temperature reaction systems. Addressing these challenges requires the development of catalytic systems featuring efficient promoters and supports to enable selective HDO and thereby reduces the demands of the reaction.

A variety of hydrotreatment catalysts can be used for hydrodeoxygenation. The review discusses six major groups of selective catalysts, including metal sulfides, transition metals (free-sulfur catalysts), and metal phosphides, nitrides, carbides, and oxides. 

### 6.2. Sulfided Catalysts

Metal sulphide (MS) catalysts catalyzed the HDS and HDN reactions in petroleum refining [89]. Because they have a high H_2_ sticking probability and can activate hydrogen, these catalysts have historically been used in the catalytic hydrogenation of model compounds derived from bio-crude oil or lignin [113].

One common type of MS catalyst is the supported sulfide catalyst, typically supported on materials like alumina or zeolites, for example (Al_2_O_3_, TiO_2_, zeolites, etc.). While effective for hydrotreatment processes, they tend to deactivate rapidly when used with water-containing heavier feeds. This is mainly due to steric hindrances within the porous support structure, leading to coking, and the transformation of the active phase due to the degradation of the support material under high-temperature water treatment [114].

Dispersed and unsupported sulphide catalysts are the new types of MS catalysts that are currently the focus of research. This is because, the pore-plugging issue is resolved by using dispersed catalysts for hydrocracking and upgrading crude bio-crude oil instead of their conventional alumina or zeolite-supported counterparts [101,115]. 

Effective dispersion of catalytic precursors within oil feeds significantly improves diffusion efficiency, resulting in the formation of highly active systems that exhibit enhanced resistance to coke formation. This is because the dispersed catalyst acts as an efficient agent for hydrogen transfer, effectively suppressing the generation of organic radicals. In the case of conventional sulphided catalysts supported on alumina or aluminosilicates, a notable challenge arises when reactants with high molecular weight exceed the pore size of the support [112]. 

The addition of promoters to Mo-based sulphide catalysts is another efficient method to improve the activity and selectivity of sulphide catalysts in HDO. Shabtai et al. [116] conducted a comprehensive study, contrasting the rate constants for aromatic ring hydrogenation (like naphthalene) and diphenyl ether’s CO bond hydrogenolysis. The promotion of C–O bond hydrogenolysis followed the sequence Ru > Co > Cr > Ir > Re > > Pd > Fe > Rh > Pt > Ni. The selectivity was influenced by various factors including the nature, concentration, preparation method, and sulfiding conditions of the promoter. Cobalt or Nickel is often chosen as a promoter on MoS_2_ due to its capability to form an active Co–Mo–S phase, considering both cost-effectiveness and activity enhancement [117,118]. 

Sulfides stand out as highly reported catalysts with remarkable arene selectivity in terms of catalytic performance. For instance, Victoria et al. [119] achieved a 73% arene selectivity using a MoS_2_ catalyst in the HDO of cresol at temperature of 325 °C and 41 bar hydrogen pressure. Wang et al. [120] showed how surfactants affect the arene selectivity of MoS_2_ catalysts, and also proved that MoS_2_ layer stacking was altered by various surfactants, with polyvinylpyrrolidone (PV) surfactant leading to significantly higher arene selectivity (>90%) in HDO of cresol at temperature of 300 °C and 40 bar hydrogen pressure.

Hydrothermal synthesis of unsupported catalysts like CoMoS and NiMoS is common, but these catalysts are often modified to be bi-functional by adding a second metal with lower reactivity, such as Mo or Fe [79]. Bunch et al. [121,122] analysed the benzofuran reaction network in HDO over the sulfided NiMo/Al_2_O_3_ catalyst. The hydrogenolysis reaction pathway continued to be predominant in the sulphide form even after the introduction of nickel.

However, effectively addressing deactivation is a critical challenge when utilizing sulfides, and this deactivation can be induced by oxygen-containing molecules that are present in both the feedstock and the resulting products. Mo’s high affinity for oxygen can result in the substitution of oxygen for sulphur, forming less active oxides and ultimately causing catalyst deactivation [123]. To counter this, maintaining the catalysts’ reactivity and sulphide form requires co-feeding a sulphide additive [124].

Gutierrez and colleagues [125] conducted a study using sulfided CoMo and Ni-Mo catalysts to hydrodeoxygenate guaiacol. They discovered that the methoxy group in guaiacol could react with SH- to produce compounds that contained sulphur, with phenol exhibiting the least amount of S-leaching. However, conventional hydrotreating catalysts lack the potential for high-arene selectivity [89] and confront issues with coke accumulation, product contamination with sulphur, and deactivation caused by sulphur loss [126]. These obstacles represent significant hindrances to their broader application in HDO [127].

Importantly, these catalysts have limitations due to the low-sulfur content in bio-crude oil. During HDO processing, MS catalysts tend to lose sulfur. To prevent catalyst oxidation and maintain the sulfur phase, a continuous supply of a sulfurizing agent, typically H_2_S, is needed [128]. In addition to oxygen, water produced during the HDO process oxidizes sulphide species, resulting in deactivation [101,115]. Moreover, the choice of support is critical; for instance, Al_2_O_3_ can convert into boehmite (AlO(OH)) due to the involvement of oxygen-containing compounds, and its high acidity can lead to surface carbon formation, reducing its lifespan [123].

The deactivation of MS catalysts in the deoxygenation of raw bio-crude oils has been extensively studied. Sulphide catalysts have garnered significant attention in the literature when it comes to deactivation and are recognized as being more established in this aspect [112]. Table 4 summarizes the MS catalysts investigated in the HDO reaction, along with other relevant data, while Table 5 presents a concise overview of the advantages and limitations of this type of catalysts.

## 7. Transition Metal Catalysts (Sulfur-Free Catalyst)

Conversely, due to the near absence of sulfur in bio-crude oil, catalysts based on transition metal sulfides tend to undergo conversion to their oxide form [38,111]. Consequently, increasing the stability of metal sulfide catalysts during hydrotreatments of bio-crude oils is a critical challenge.

The initiative to use both monometallic and bimetallic noble/non-noble transition metal catalysts in HDO reactions offers a potential solution. These catalysts are intended to achieve a higher degree of deoxygenation than commercial MS catalysts, without the need for a sulfur co-feed to prevent deactivation, and they show a higher resistance to poisoning during operation [60]. However, it is advised to remove sulfur-containing compounds from bio-crude oil before putting it through HDO treatment because transition metals, especially the noble metals, are more sensitive to sulphur [30].

### 7.1. Monometallic Catalysts

Transition metals, encompassing both noble and non-noble varieties, have garnered significant interest in the deoxygenation of compounds within bio-crude oils due to their exceptional hydrogen activation capabilities [140].

Among transition metals, noble metal transition (NMT) catalysts, such as Pd, Pt, Rh, Ru, and others, are prominently featured. Notably, Rh, Pt, and Pd, when supported on less acidic bases like ZrO_2_, exhibit high activity and stability, making them viable alternatives to the conventional sulfide CoMo/Al_2_O_3_ catalyst [141]. Research by French et al. highlighted that, even at high H_2_ pressures (70–170 bar), NMT catalysts still resulted in oxygenated aromatic compounds when compared to conventional sulfide NiMo catalysts. As the temperature increased from 340 to 400 °C, phenolic compounds transformed into aromatics and cycloalkanes [38]. NMT catalysts, particularly those with Pt, demonstrate high catalytic activity in hydrogenation. Often, researchers combine Pt with different carriers to maximize the synergy between the carrier’s properties and Pt’s deoxidizing capabilities [142]. For instance, the degree of deoxygenation increases when Pt is combined with the acidity of zeolite. However, Pt-based catalysts do have a notable drawback, which is lower product yield [143].

NMT configurations show promise for long-term application in the HDO process for phenolics. In early glycerol hydrogenolysis research, ruthenium (Ru)-based catalysts were prominent [144]. Feng et al. [145] investigated the influence of various supports (TiO_2_, SiO_2_, NaY, -Al_2_O_3_) on Ru-based catalysts. TiO_2_-supported catalysts exhibited the highest activity, achieving a glycerol conversion rate of 90.1%. However, this catalyst favored ethylene glycol production over 1,2-propanediol (1,2-PDO). Conversely, Ru/SiO_2_, with lower activity, displayed significantly higher selectivity for 1,2-PDO. Importantly, the choice of support affected the size of the Ru particle, with smaller Ru particles enhancing catalyst activity.

Beyond Ru, other noble metals have also undergone extensive investigation. For example, Furikado et al. [146] assessed the performance of diverse noble-metal catalysts (Rh, Ru, Pt, and Pd) supported on materials like carbon (C), SiO_2_, and Al_2_O_3_. Rh/SiO_2_ catalysts yielded the highest selectivity for 1,2-PDO, especially at lower reaction temperatures and glycerol conversions. However, these catalysts demonstrated lower selectivity toward 1,2-PDO due to excessive over-hydrogenolysis of 1,2- and 1,3-PDO to 1,2-propanediol and 2-propanol.

Crucially, catalysts prepared through varied techniques exhibit significant distinctions. Shu et al. [147] observed that the polyol reduction method resulted in a better pore structure and Ru dispersity (13.32%) compared to the impregnation method (Ru dispersity of 9.63%). The former method produced smaller Ru crystal sizes, generating more acidic sites. This study highlighted the new catalyst’s remarkable hydrocarbon selectivity (86.1%). NTM catalysts significantly enhance the deoxygenation process, but they are costly and less efficient reagents for solid waste-based reuse [38]. Also, they exhibit higher hydrogenation activity compared to NNTM catalysts. However, due to the propensity of benzene rings in the reactants to become saturated during the HDO process, NTM catalysts alone are not ideal for the production of aromatic hydrocarbons.

On the other hand, non-noble transition metal (NNTM)-based catalysts, including Ni, Co, Fe, Mo, V, W, and Cu, have been extensively studied for hydrodeoxygenation (HDO) due to their strong reduction capabilities [112]. NNTM catalysts, especially Co, Fe, and Mo, often favor the direct deoxygenation (DDO) route, resulting in the creation of aromatic hydrocarbons [89]. Nickel, with its excellent hydrogenation activity and lower electrophilicity compared to metals like molybdenum (Mo), has received significant attention in HDO processes. It shows high activity for hydrogenating aromatic rings, reducing its susceptibility to direct activation of the C=O and C–O bond [134,148]. Mortensen et al. [101] investigated a large number of NNMT-based catalysts, and the results showed that nickel-based catalysts produced the best results in the HDO reaction with phenol.

Cobalt (Co) is another prominent NNMT recognized for its efficacy in removing oxygen through the DDO route, outperforming other transition metals like Ni in synthesizing aromatic hydrocarbons [26,149]. It is primarily utilized as a promoter within various catalytic systems, rather than being considered an active phase by itself. Unlike Ni, which is a common choice for monometallic noble metal-free catalysts in HDO processes, Co is not typically viewed as a standalone active component [62,149]. Given its abundance and affordability in comparison to other transition metals, iron (Fe) presents itself as a desirable option for the catalyst role in HDO processes for the refining of bio-crude oil [150]. Fe shows promise in the HDO of phenolics, showing a strong preference for deoxygenated aromatic compounds despite being conventionally thought of as being unreactive in the hydrogenation of aromatic rings. Even though they might be less competitive in deoxygenating phenolics than catalysts containing Ni or Co, Fe-based catalysts may help the HDO process save hydrogen [150]. Olcese et al. reported that Fe-based catalysts showed selectivity towards aromatic compounds in the HDO reaction of guaiacol [151]. Zinc (Zn), acting primarily as an auxiliary in the process, displays less activity compared to other transition metals [62].

Using NNMT catalysts does have limitations. While they’ve shown good bio-crude oil HDO yields, enhancing their reduction capacity is crucial to efficiently catalyze the HDO reaction without needing extra reduction steps when the active phase oxidizes [73]. Table 4 provides a summary of some NNMT and NMT catalysts studied in bio-crude oil HDO processes, while Table 5 outlines their advantages and disadvantages.

### 7.2. Bimetallic Catalysts

**Figure 10:**
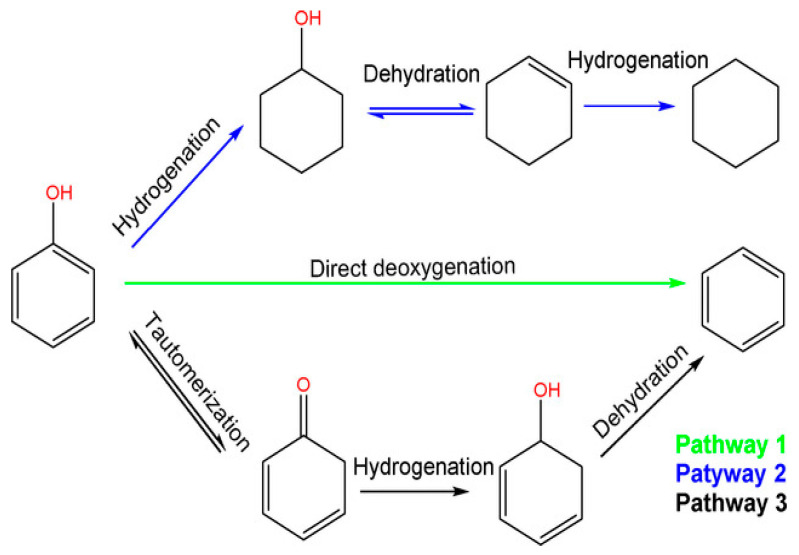
DDO, HYD, and tautomerization-deoxygenation are three potential reaction pathways in the HDO process of phenol [89].

Bimetallic catalysts have gained significant attention in recent years for deoxygenating phenolic compounds. Their development was intended to improve the stability of monometallic catalysts by increasing their activity towards DDO pathways rather than HYD and ring-opening reactions as shown in Figure 10 [89]. Combining active metals has been the focus of research like Pd, Pt, and Ni with less active metals such as Cu, Co, Sn, and Fe within the past three years [139]. The proposed solution involves modifying the selectivity of the catalyst by adding secondary metals, favoring DDO reaction pathways over HYD ones.

Compared to monometallic catalysts, bimetallic catalysts offer a more effective approach because the interaction between the metals can enhance selectivity to specific products by altering the metal surface’s geometric and electronic structures [139].This formulation shows promise for achieving high catalytic activity with relatively mild reaction conditions and minimal hydrogen consumption compared to monometallic systems. Some authors have concluded that the increased HDO activity over bimetallic catalysts is related to improved demethoxylation and deoxygenation pathways [62]. 

For instance, numerous studies have been conducted on the NiCo bimetallic catalytic system for upgrading phenolic compounds or bio-crude oil derived from lignin. NiCo bimetallic catalysts demonstrated superior performance compared to monometallic Ni and Co catalysts, which is attributed to the presence of a NiCo alloy, which increased the stability and dispersion of the Ni active phase [152]. Similarly, alternatives like PtCo, NiPd NiCu, and others showcased heightened HDO activity, comparing HDO activity to that of their monometallic counterparts [89,153]. However, achieving high deoxygenation degrees in these catalytic systems generally requires the presence of acidic sites.

To increase HDO activity and alter the distribution of products, two main strategies have been used. The first method makes use of oxyphilic metal (OM) catalysts, which are composed of oxyphilic metals like Re, Fe, and Mo along with noble or base metals like Pd, Pt, Ru, and Ni. By using this method instead of acidic supports, HDO activity and product distribution are improved. By avoiding associated drawbacks like promoting side reactions like polymerization in bio-crude oil HDO and minimizing rapid catalyst deactivation due to coke formation, this method outperforms metal-acid support catalysts [154]. In the Huynh et al. study [1], it was shown that Ni-Co catalysts improve ring-saturation reactions, showing that the incorporation of Co into Ni ineffectively promotes the DDO pathway. This is because the incorporation of Co into Ni causes a decrease in the size of Ni particles, increasing conversion rates and helping to prevent catalytic deactivation caused by coke deposition.

Catalysts with alkali metals (Na, K) are used in the second strategy. Dopants like Na and K can reduce deactivation problems in catalyst formulations, particularly for metal-acid catalysts. These alkali metals change the acidity of the acid support through interaction. For instance, K’s acidity-modifying properties can enhance the deactivation resistance of CoMo/Al_2_O_3_ catalysts [137]. The selectivity was changed, which shifted it away from direct cleavage of an aromatic compound’s C–OH bond, hydrogenation reactions and toward demethylation and methyl substitution reactions.

Bimetallic catalysts like NiPt and CoPt supported on Al_2_O_3_ have been shown in studies to have higher activity than monometallic Pt catalysts for the HDO of *m*-cresol [155]. By creating new acidic sites through the incorporation of transition metals, the selectivity for saturated cyclic hydrocarbons was increased. Further studies on PtMo catalysts with 5% loading of Pt supported on Al_2_O_3_ showed that Mo increased deoxygenation production in the *m*-cresol HDO reaction in comparison to Pt monometallic catalysts [156]. The synergistic effect was clearly seen when Mo was added as a second metal to noble metal catalysts, which significantly increased conversion and selectivity. 

**Figure 11:**
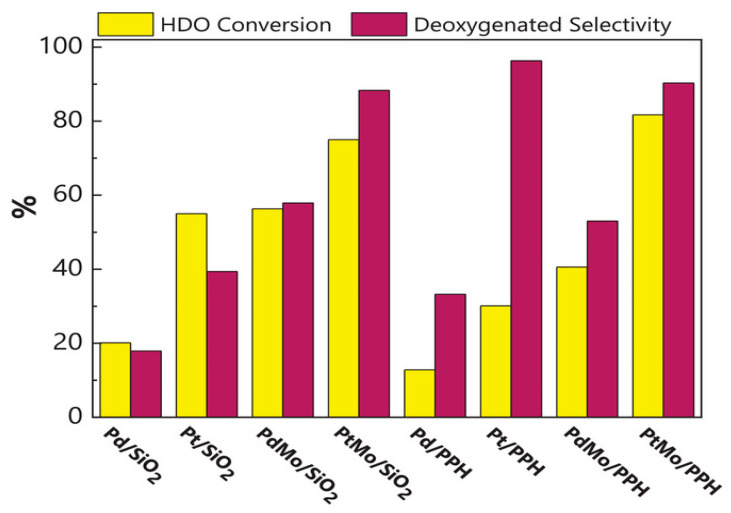
DBF’s deoxygenated selectivity over bifunctional catalysts and conversion [157].

A comparison of catalysts made of either monometallic or bimetallic combinations of 1% weight Pt or Pd and 1% weight Mo is shown in Figure 11 [157]. As the model molecule, dibenzofuran (DBF) was used, and the experiments were carried out at 15 bar and 275 °C. The results highlight the promoter function of molybdenum, which significantly improves HDO activity with both Pd and Pt catalysts over several hours of reaction time without catalyst deactivation. This demonstrates that using noble metals produces catalysts for hydrotreating reactions that are more effective than those that are typically based on molybdenum sulfide. The research study also highlights the possibility of improving the catalytic performance of noble metals by utilizing mesoporous supports with Lewis acidity and a synergistic second metal, which aids in promoting the hydrogenolysis reaction. These catalysts are more suitable for industrial applications due to the possibility of using a smaller amount of noble metal in the catalyst structure. Table 4 summarises important bimetallic catalysts investigated in the HDO model compounds that were discovered in pyrolyzed biomass reacting. 

## 8. Transition Metal-Based Catalysts, Oxides, Phosphides, Carbides, and Nitrides

Metal carbides, nitrides, and phosphides are highly effective in various hydrogen-related reactions, like CO hydrogenation, ammonia synthesis, and neopentane hydrogenolysis. They blend the features of both metals and ceramics, showcasing remarkable catalytic activity [158].

Due to the high cost and limited availability of noble metals, researchers are actively exploring alternative, more cost-effective catalytic materials. Transition metals, also known as NNMT (non-noble metal catalysts), have emerged as promising alternatives, whether in their metallic state or as oxides, carbides, nitrides, or phosphides. NNMTs have several advantages: they are abundant, cost-effective, sulfur-free, and possess an oxyphilic nature.

When non-metallic atoms are introduced into a metallic lattice, they alter the electronic structure, resulting in a unique configuration. This, in turn, reduces the density of active sites on the catalyst’s surface. Consequently, adjusting the ligand-to-metal ratio allows for the modification of the catalytic properties of transition metals [101]. Table 5 presents a comprehensive comparison of different catalysts, outlining their key characteristics.

### 8.1. Metal-Oxide Catalysts

Metal-oxide (MO) catalysts have garnered considerable attention due to their potential in various catalytic processes. Some MO catalysts are believed to exhibit selectivity for direct deoxygenation (DDO) due to the presence of oxygen vacancies, which act as acid sites. Notably, MO catalysts like Mo, Ni, W, and V have been reported in the literature for their significant catalytic activity in the HDO reaction. The catalytic activity of oxides in HDO is generally determined by the acidic sites they possess.

Bronsted acidity has an impact on the availability of acidic sites on the oxide catalyst during the first stage of chemisorption, where the oxygen lone pair of oxygenated compounds can chemisorb [1]. However, to stop active species from becoming inactive, a low H_2_ pressure must be maintained., aligning with the fundamental mechanism of oxide catalysts.

Prasomsri et al. [159], have substantiated this notion by utilizing MoO_3_ catalysts to deoxygenate a range of model compounds derived from biomass. Their work demonstrated the successful conversion of aromatic oxygenates into oxygen-free aromatics, affirming the catalyst’s promotion of the DDO chemical pathway. Addressing catalyst deactivation can be achieved through a straightforward calcination process. Furthermore, elevating the H_2_ pressure is recommended to mitigate the hindrance caused by water at active sites; however, this approach may not be feasible in systems aiming to minimize hydrogen consumption. 

Metal-oxide (MO) catalysts have attracted significant attention due to their potential in various catalytic processes. Some MO catalysts are known to exhibit selectivity for direct deoxygenation (DDO) owing to the presence of oxygen vacancies, which act as acid sites. Notably, catalysts like Mo, Ni, W, and V based on metal oxides have shown substantial catalytic activity in the hydrodeoxygenation (HDO) reaction. The catalytic activity of oxides in HDO is primarily determined by the presence of these acidic sites.

The Bronsted acidity of an oxide catalyst significantly influences the availability of acidic sites during the initial stage of chemisorption, where oxygenated compounds chemisorb through their oxygen lone pairs [138]. However, to prevent active species from becoming inactive, maintaining a low H_2_ pressure is crucial, aligning with the fundamental mechanism of oxide catalysts. Prasomsri et al. [151] provided evidence supporting this idea by utilizing MoO_3_ catalysts to deoxygenate various model compounds derived from biomass. Their research demonstrated the successful conversion of aromatic oxygenates into oxygen-free aromatics, confirming the catalyst’s promotion of the DDO chemical pathway. Addressing catalyst deactivation can be achieved through a simple calcination process. Additionally, raising the H_2_ pressure is advisable to mitigate hindrances caused by water at active sites. However, this approach may not be feasible in systems aiming to minimize hydrogen consumption.

### 8.2. Metal-Phosphide Catalyst

Transition metal phosphide (TMP) catalysts represent a robust and relatively new catalyst type proposed for the catalytic oxygenation of biocrude oil compounds, which has recently gained attention due to acidic H_2_-active sites in HDO [1]. TMP catalysts have been studied for their ability to form HDO catalysts, including FeP_x_, CoP_x_, NiP_x_, MoP_x_, WP_x_, and bimetallic phosphides [101]. Moreover, TMP catalysts exhibit catalytic ability similar to noble metals such as Pt in hydrocarbon dehydrogenation and hydrogenation, exhibiting exquisite hydrogen transfer ability.

It is worth noting that TMP catalysts facilitate decarboxylation, whereas metal catalysts promote demethylation. Catalysts containing phosphorus (P) are especially effective for bio-pyrolysis oils that contain a high concentration of carboxylic acids. Both components of these catalysts effectively enhance carboxylic acid conversion and deoxygenation. For example, Mendes et al. [160] observed high decarboxylation activity in phosphides, mainly by removal of oxygen as CO_2_, highlighting their decarboxylation efficiency.

Moreover, Ni phosphides in different valence states fill micropores, enhancing decarboxylation due to the better CO adsorption capacity, due to Ni_12_P_5_ as compared to other phosphides. In a comparable study, Gonçalves et al. [161] found that nickel phosphide as a support of SiO_2_ and ZrO_2_ exhibited greater activity than metallic nickel of cresol HDO. Another study, by Zhao et al. [162], studied guaiacol HDO using SiO_2_-based catalysts including Ni, Mo, Co, Fe, and W phosphides. Among the transition metals, Ni exhibited exceptional hydrogenation activity, resistance to coke formation and weak acidity.

In contrast to NMT and NNMT, the addition of P will result in the production of additional active materials and crystal phases, as well as an increase in the catalyst’s dispersion and the transition metals’ capacity to withstand acids. But as more P is added, its activity will decline, and P-doping will unavoidably harm the pore structure. Therefore, the problem of how to effectively utilize P’s benefits remains an area of concern [1].

### 8.3. Metal-Carbide Catalyst

Metal-carbide catalysts, especially tungsten and molybdenum carbides, have emerged as promising substitutes for sulphide, oxide, and phosphide catalysts in the deoxygenation of compounds found in bio-crude oil. To facilitate carburization without requiring methane, these carbides are often supported on carbon supports [1]. Notable examples of carbides include SiC, CaC_2_, and WC, representing covalent, ionic, or interstitial carbides. Among these, interstitial carbide catalysts are the most commonly used [38].

In the 1970s, Levy et al. [157] noted that tungsten’s electronegativity changed with the formation of tungsten carbide, causing this new species to act like platinum in catalyzing the reaction of H_2_ and O_2_. This discovery sparked significant interest and subsequent investigation of carbide catalysis, driven by carbon’s influence on the metal’s d-band occupation and Fermi level. Despite favoring reaction pathways without ring saturation and reducing H_2_ consumption, carbide catalysts have limitations, including the need for high temperatures (around 1000 °C) for the carburization process, and irreversibility upon deactivation, making proper regeneration challenging [15]. Jongerius et al. [158] investigated the deoxygenation of guaiacol using W_2_C and Mo_2_C catalysts and found them to be mostly selective for phenol production via de-methoxylation, with few ring-saturation reactions at 55 bar H_2_ and temperatures ranging from 300 to 375 °C.

Their catalytic effectiveness in hydrotreating reactions is akin to that of noble metal catalysts, exhibiting a strong affinity for breaking C–O/C=O bonds. This leads to enhanced selectivity towards aromatic compounds. Moreover, incorporating a second metal further enhances selectivity and catalytic conversion by forming a solid solution with the primary metal. For instance, Smirnov et al. [163] examined the HDO reaction and found that nickel and molybdenum carbides favor the decarboxylation pathway while minimizing coke deposition. Also, bimetallic molybdenum and tungsten carbides demonstrate heightened catalytic activity in the HDO of guaiacol due to increased exposure of metallic active sites capable of activating hydrogen molecules.

Catalysts based on molybdenum show significant promise for the conversion of lignin-derived bio-crude oil into aromatic hydrocarbons under atmospheric pressure. However, in the presence of H_2_O, Mo_2_C catalysts can be deactivated as water oxidizes Mo_2_C to MoO_2_, which exhibits lower HDO activity. Therefore, efficient removal of H_2_O generated during the HDO process is crucial [164]. Additionally, MoO_3_ catalysts supported on ZrO_2_ and TiO_2_ significantly enhance toluene selectivity compared to unsupported MoO_3_ catalysts [164].

### 8.4. Metal-Nitride

Transition metal nitrides share intriguing characteristics, resembling both ceramics and noble metals, making them attractive catalysts for HDO, even though they may be less selective for producing oxygen-free products. The active species in these catalysts coordinate unsaturated metal atoms and nitrogen vacancies, enabling unconventional yet favorable HDO reaction routes [111]. 

In the hydrodeoxygenation of guaiacol, for instance, when using carbon-supported MoN, there was a total conversion exceeding 50%, showcasing strong selectivity towards phenol and catechol. This selectivity arises from demethoxylation occurring at unsaturated Mo sites and demethylation at sites with low levels of nitrogen [1]. Nitride catalysts, despite fewer studies compared to carbides and phosphides for HDO, have shown superiority over water-sensitive carbide catalysts, even though water is expected to be present during HDO treatment [38].

While fewer studies have been conducted on nitride catalysts compared to carbides and phosphides for HDO, various nitrides have been reported, including MoNx, WN, VN, NbN, and TiN [111]. However, conventional pyrolysis techniques using metal halides or metal-organic precursors, commonly used for transition metal nitrides, are unsuitable as they do not result in the desired porous structure necessary for efficient HDO reactions [77].

## 9. Coke Formation

In HDO reactions, the principal contributors to catalyst deactivation include coke deposition, metal leaching, sintering, poisoning, and surface oxidation resulting from exposure to water. 

Because coke formation affects catalyst activity and selectivity due to the deposition on active sites, as well as because carbon is a byproduct of both polymerization and polycondensation reactions that occur in the HDO reaction as a result of the high temperature and pressure effect. The extent and type of the coke deposit are also determined by the size, shape, and distribution of the pores and the crystals [38].

The formation of coke during catalytic processes is influenced by multiple factors. Notably, increased catalyst acidity correlates with higher coke formation. For example, alumina, possessing high acidity, deactivates faster compared to inert supports like SiO_2_ due to strong interactions with coke precursors [137]. A high density of Bronsted acid sites accelerates both coke formation and precursor condensation. Therefore, a balance between catalyst activity (linked to acidity) and managing deactivation is crucial [165].

The second reason is that different feedstock types also have a big impact on coking deposition. This is because alkenes and aromatics, which are unsaturated hydrocarbons with a high coking potential interact strongly with the C=C bond and aromatic rings on the metal active catalyst sites [158]. It was found that oxygenated compounds with more than one oxygen atom have a stronger capacity for carbon to form through polymerization reactions [38]. Moreover, finding a solution is crucial, and this can be achieved through the process of regeneration. 

Calcination is a commonly employed technique in which coke deposits on the catalyst surface are removed under an oxidant atmosphere at temperatures reaching up to 600 °C. Another approach for catalyst regeneration involves using a solvent, but this method generates numerous acid sites, potentially reducing catalyst activity. A more promising regeneration method involves oxidation, wherein catalysts undergo oxidation and/or reduction with H_2_ gas to produce a highly active catalyst without the need for developing a new one, ultimately reducing overall cost [38].

However, there are numerous other ways to prevent coke formation in the HDO process, including the use of co-feed bio-crude oil and this is carried out by the addition of a hydrogen donor solvent like decalin, propanol, tetralin, or methanol. The primary purpose of the hydrogen donor solvents is to dilute lignin, which stops coke from polymerizing at high reaction temperatures. However, the reaction also increases selectivity by promoting the hydrogenation and cracking reactions [166]. Patil et al. [167] investigated tetralin as a hydrogen donor solvent and studied guaiacol’s HDO at 330 °C for 15 to 600 min with 30 bar of H_2_ pressure. Guaiacol showed complete conversion over the 600 min period, resulting in 45.3% phenol and 11.1% cyclohexane as the final products.

In summary, according to Cheng et al. [165], there are a number of ways to stop coke from forming during the HDO treatment, including using a catalyst support with low acidity, like silica and activated carbon; conducting the HDO reaction under mild conditions; carrying out the reaction in multiple steps rather than just one HDO step; and adding hydrogen donor solvents to increase the dilution. 

## 10. Catalyst Regeneration

Regeneration and recycling of the deactivated catalyst following the HDO reaction are essential from an economic standpoint in order to lower the overall cost. Coke oxidation in a stream of air at an appropriate temperature, typically between 350 and 600 °C, is one of the most popular methods.

The process involves three key steps: Firstly, the catalysts with adsorbed organic species are washed and dried. Next, the dried catalysts undergo calcination in an oxy-gen-containing gas like air. Lastly, the calcined catalysts are reduced in a hydrogen-containing gas [165]. For example, Pt/H-MFI-90 coke deposition catalysts are cleaned with ethanol and methanol, then calcined in air at 400 °C for 10 h, and finally reduced in a continuous hydrogen gas flow. Additionally, the CoMo/MCM-41 HDO catalyst is regenerated by being exposed to air for two hours at 600 °C. It was observed that although the regenerated catalyst had lower hydrogenation activity due to reduced surface area and pore volume compared to the fresh catalyst, both produced comparable yields of bio-crude oil. Similar results when Ni-Cu/ZrO_2_-SiO_2_ catalyst was generated through ethanol washing for guaiacol HDO at 300 °C and 5.0 MPa hydrogen pressure; it was found that after the fourth recycle, guaiacol conversion and benzene selectivity were significantly reduced due to coke formation and residual polymer deposits on the catalyst surface [8].

However, a different study found that the use of Pt/-Al_2_O_3_, Pt/-SiO_2_, and Pt/-Na-B for upgrading of cresol, where three catalysts were regenerated in air at 350 °C, had the same impact on activity and selectivity for toluene as the first run. This was primarily because of the recovered all-metal sites and the high proportion of acid sites [168].

Addressing the challenge of coke removal from an activated carbon (AC)-supported catalyst, air combustion is a preferred method. Recycling used AC-supported catalysts is a practical approach. For example, the reused Ru/C catalyst undergoes cleaning with ethanol. However, with increased recycle times, the catalyst’s selectivity towards desired products progressively declines due to coke deposition [169]. 

## 11. Conclusions

Researchers have dedicated their efforts to improving the quality of bio-crude oil obtained through the fast pyrolysis of biomass. This is crucial because high oxygen content is a primary factor diminishing the chemical and physical properties of bio-crude oil for conventional applications. This review provides a comprehensive analysis of various upgrading techniques aimed at producing oxygen-free bio-crude oil. After considering environmental, and economic factors, it becomes evident that hydrotreating emerges as the most promising method for biofuel applications. Meanwhile, solvent addition offers a cost-effective route to achieving the desired low viscosity of bio-crude oil. Most of the current upgrading techniques hold great potential for generating bio-crude oil as a preferable alternative to conventional petroleum-derived fuels. Additionally, this review explored different types of catalysts used in hydrodeoxygenation (HDO) and observed that noble metal catalysts enable reactions at significantly lower pressure and temperature levels, ultimately reducing production costs. On the other hand, non-noble transition metal oxides, nitrides, phosphides, and carbides, offer the advantages of abundance and lower cost. However, their careful utilization is essential to achieving the necessary reduction power for HDO reactions on oxygenated bio-crude oil molecules and to addressing issues arising when the active phase becomes oxidized. An encouraging approach could involve the use of bimetallic catalysts that combine noble metals with non-noble components, considering the distinctive characteristics of both. This strategy proves advantageous when considering both catalyst performance and economic factors. Bimetallic catalysts tend to be selective for arenes (BTX), which reduces hydrogen gas consumption and increase the yield of hydrocarbons. This review highlights the potential for such innovative catalysts to play a pivotal role in the sustainable production of oxygen-free bio-crude oil, contributing to a cleaner and more sustainable energy future.

## Figures and Tables

**Table 1:** Common compounds in bio-crude oil derived from wood [9].

Different Groups Compounds	Categories of Functional Groups	Typical Compounds
Water	-	Water
Simple oxygenates (non-aromatic)	Esters	Methyl acetate, ethyl acetate, methyl formate
	Alcohols	Methanol, Butanol, Cyclohexanol, Cyclopentanol, ethylene glycol
	Acids	Hexanoic acid, Acetic acid, Propanoic acid, Formic acid, Butanoic acid, glycolic acid
	Aldehydes	2-butenal, glyoxal, formaldehyde, benzaldehyde
	Ketones	Cyclohexanone, Cyclopentanone, 2-butanone
Sugars and sugar derivatives		Xylose, glucose, fructose, sorbitol, arabinose
		Levoglucosan
Furans	-	Furfural, furfuryl alcohol, tetrahydrofuran, 2-furanone, butyrolactone, 2-acetyl furan, methyl-2-furanone, 2,5-dimethyltetrahydrofuran
Aromatics	Oxygen free	Toluene, Benzene, Xylene
	Phenols	Methylphenol, dimethylphenol, ethylphenol, phenol
	Catechols	Methylcatechol, ethylcatechol, methoxycatechol, catechol
	Guaiacols	Methylguaiacol, ethylguaiacol, eugenol, vanillin, guaiacol
	Syringols	Methylsyringol, ethylsyringol, 4-propenylsyringol, syringol
Miscellaneous oxygenates	-	2-hydroxyacetaldehyde 1-hydroxy-2-propanone 1-hydroxy-2-butanone
High molecular weight species	-	Dimers, trimers, oligomers and cellulose, hemicellulose, and lignin pyrolysis products

**Table 2:** Properties and element content (wt%) of bio-crude oil from fast pyrolysis and heavy oil [9,33].

Properties	Bio-Crude Oil ^(a)^	Heavy Oil
pH	2–4	-
Density (kg/m^3^)	1200	940
Moisture content (i.e., water) (wt%)	15–30	0.1
HHV(MJ/kg)	16–19	40
Viscosity (cP, 50 °C)	40–100	180
Flash point (K)	300–370	327
Solids(wt%)	0.2–1	1
Ash (wt%)	0–0.2	0.1
NO_x_ emission	below 0.7	1.4
SO_x_ emission	0	0.28
H/C	0.9–1.5	1.5–2.0
C/O	2–3.3	-
Element Content (wt%)		
Carbon (C)	48–65	83–86
Hydrogen (H)	5.5–8	11–14
Oxygen (O)	30–55	below 1
Nitrogen (N)	0–0.3	below 0.3
Sulfer (S)	below 0.05	below 3.0
Na^+^ (ppm)	5–500	-
Ca (ppm)	4–600	-

^(a)^ Characteristics of around 140 distinct bio-crude oil samples produced from diverse feedstocks and pyrolysis systems.

**Table 3:** Advantages and limitations of bio-crude oil upgrading processes.

Upgrading Approach	Advantages	Media and the Reaction	Limitation and Challenges	Ref.
Solvent Addition	Easy Operation and increases in bio-lower oil’s heating value, reduces density and viscosity	Alcohol: methanol, ethanol, and isopropanol.	Decrease in the bio-flash oil’s point. Unfavourable materials cannot be removed (Oxygen)	[38]
Emulsion	Easy operation, low cost, increase calorific value and decreases water content	Emulsifiers used—Span 20, Span 80 and Span 100. Span 85, Tween 85, Span 60, Brij 72 and hypermer B246. Tween 80, and Lignin	Surfactant costs are considerable, energy use is high, corrosion and stability issues and unfavourable chemicals cannot be removed.	[38]
Hydrotreatment (HDO)	Utilizing compressed hydrogen to remove oxygen, increasing heating value and lowering bio-crude oil viscosity, moderate reaction condition	sulfide Fe, Co, CoMo, NiMo, NiCu, NiMo/Al_2_O_3_ and CoMo/Al_2_O_3_ platinum, rhodium, ruthenium and nickel	High-pressure hydrogen is necessary, coke formation, catalyst deactivation, high cost and inefficient use of hydrogen	[62]
Steam reforming	The main product is highly energy dense H_2_.	Base and noble metal catalysts: Co, Cu, Ir, Ni, Rh, Pt, Pd and Fe. Supported materials: MgO, MgO-Al_2_O_3_, CeO_2_, ZrO_2_, Calcite, Dolomite, Al_2_O_3_ Zeolites-Y, ZnO,	Coking and catalyst cost, relatively short catalyst stability and reactivity, and the need for high-temperature-resistant reactors	[63]
Supercritical fluids	It helps bio-crude oil have a higher heating value while also reducing its acid number	Ethanol, methanol, CO_2_ and used as solvents include and water. used catalyst: Pt/C Ni	high price of the solvent	[64]
Catalytic Cracking	Removes bio-crude oil molecules that contain oxygen while also enhancing its calorific value, viscosity, acidity, and water content	Calcined Dolomite, Na_2_CO_3_, K_2_CO_3_, catalyst with nickel ZnHZSM-5, SAPO-11, FCC, H-mordenite, Ni/SiO_2_-N, MgAPO-36, and HZSM-5 Na_2_CO_3_/Al_2_O_3_, K_2_CO_3_, MgO and Ca(OH)_2_	low-grade bio-crude oil is produced, reduced catalyst life and reactor fouling	[38]
Catalytic Hydrocracking	The formation of light components	Ni-Mo and Co-Mo sulfide catalysts over zeolites	High-pressure H_2_ production, catalyst deactivation, and coke formation are necessary, as well as the need for a reactor with high pressure tolerance.	[65]
Microwave heating	Reduced energy use yet rapid reaction rates, selective heating, easy handling	HZSM-5 aluminosilicate zeolite, K_3_PO_4_, metal oxides (such as Fe_2_O_3_ and Al_2_O_3_)	Uneven heat distribution unsuitable for scale-up, difficulties in reaction monitoring	[66]
Non-thermal plasma	Low temperatures and atmospheric pressure needed for the reaction, extend catalyst durability, resulting in a high-quality product	MnO_2_/Al_2_O_3_, Ag-Mn/HZSM-5, BaTiO_3_, AgO_x_-MnO*_x_*/SMF, MnO*_x_*/SMF, BaTiO_3_/TiO_2,_ Ag/TiO_2_, Cu-Mn/TiO_2_	Frequent maintenance is required to ensure proper functioning, high operation cost	[67]

**Table 4:** Sulphide catalysts and transition metal catalysts used in the pyrolyzed biomass HDO reaction.

Catalyst	Temperature	Pressure	Reactant (Inlet)	Type of Reactor	Conversion (%)	Ref.
(a) Sulphide catalysts						
NiMo/Al_2_O_3_	350	75	Phenol	Batch	55	[129]
NiMoS	220	50	Phenol	Batch	84	[118]
NiMoS	350	28	Phenol	Batch	96	[118]
NiS	350	28	Phenol	Batch	35	[118]
MoS_2_	300	40	4-Methylphenol	Batch	52	[130]
MoS_2_	300	40	Guaiacol	Fixed bed	100	[62]
CoMoS	275	40	*p*-cresol	Batch	100	[131]
CoMoS	300	40	Guaiacol	Fixed bed	95	[62]
CoMo/Al_2_O_3_	360	70	*p*-cresol	Batch	82	[132]
(b) Monometallic transition metal catalysts						
Ni@*γ*-Al_2_O_3_	300	5	Phenol	batch	100	[133]
Ni@SiO_2_	300	5	Phenol	batch	99	[133]
(c) Bimetallic transition metal catalysts						
Ni-5Fe@CNT	400	3	Guaiacol	Fixed-bed	47.2	[134]
5Ni-Fe@CNT	400	3	Guaiacol	Fixed-bed	96.8	[134]
CoMo	400	2.8	Guaiacol	Fixed-bed	88.5	[135]
NiMo	400	2.8	Guaiacol	Fixed-bed	99.9	[136]
NiMoW	400	2.8	Guaiacol	Fixed-bed	99.6	[136]
CoMo	250	5.5	Guaiacol	Batch	100	[137]
(d) Phosphide Catalysts						
CoP@SiO_2_	300	3	Phenol	Fixed bed	98	[138]
Co_2_P@SiO_2_	300	3	Phenol	Fixed bed	23	[138]
CoP_2_@SiO_2_	300	3	Phenol	Fixed bed	99	[138]

**Table 5:** Advantages and disadvantages of various types of HDO catalysts.

	Advantages	Disadvantages	Ref.
Sulphide Catalysts	Higher resistance to sulfur poisoning Lower cost Used widely in HDS and HDN industries	Higher deactivation rate and carbon deposition Needs continuous supply of H_2_S. Deactivated by water	[38]
Monometallic Catalysts	Higher life-spin and stable performance Lower metallic loads.	High cost especially for Noble catalysts Intolerant for sulphur in the feed and high possibility of sulphur poisoning Deactivated by water.	[1]
Bimetallic Catalysts	Promoting solvent-free HDO High HDO activity	Oxidization of transition metal	[139]
Metal-oxide Catalysts	Oxyphilic nature Good stability and performance in HDO High availability Lower cost compared to noble metals	Coke deactivation and easily oxidizable Has lower performance than the noble catalysts. Reduced and reactive active phase is needed	[1]
Metal-phosphide catalysts	More selective than metal oxide Designing the structure and acid sites for HDO is important for higher efficiency	Higher possibility of phosphide generation Choosing the synthesis method is complicated and metal loading as well.	[101]
Metal-carbide catalysts	Similar behaviour to noble catalysts High resistance to coke formation by formation of bimetallic High selectivity	Low stability	[1]
Metal-nitride	Similar behaviour to noble catalysts	Less selective to deoxygenates. Need to reduce with H_2_/N_2_ to achieve high dispersion	[111]

## Data Availability

The data presented in this study are available upon request from the corresponding author. Requesters will need to sign a data access agreement. Data can be made available starting with the date of publication of this Article and up to 5 years thereafter.
